# Electrical stimulation of non-classical photon emission from diamond color centers by means of sub-superficial graphitic electrodes

**DOI:** 10.1038/srep15901

**Published:** 2015-10-29

**Authors:** Jacopo Forneris, Paolo Traina, Daniele Gatto Monticone, Giampiero Amato, Luca Boarino, Giorgio Brida, Ivo P. Degiovanni, Emanuele Enrico, Ekaterina Moreva, Veljko Grilj, Natko Skukan, Milko Jakšić, Marco Genovese, Paolo Olivero

**Affiliations:** 1 Physics Department and “NIS” Inter-departmental Centre University of Torino; INFN Sez. Torino; CNISM Research Unit – Torino; via P. Giuria 1, 10125, Torino, Italy; 2 Istituto Nazionale di Ricerca Metrologica (INRiM); Strada delle Cacce 91, 10135 Torino, Italy; 3 Ruđer Bošković Institute, Bijenicka 54, P.O. Box 180, 10002 Zagreb, Croatia

## Abstract

Focused MeV ion beams with micrometric resolution are suitable tools for the direct writing of conductive graphitic channels buried in an insulating diamond bulk, as already demonstrated for different device applications. In this work we apply this fabrication method to the electrical excitation of color centers in diamond, demonstrating the potential of electrical stimulation in diamond-based single-photon sources. Differently from optically-stimulated light emission from color centers in diamond, electroluminescence (EL) requires a high current flowing in the diamond subgap states between the electrodes. With this purpose, buried graphitic electrode pairs, 10 μm spaced, were fabricated in the bulk of a single-crystal diamond sample using a 6 MeV C microbeam. The electrical characterization of the structure showed a significant current injection above an effective voltage threshold of 150 V, which enabled the stimulation of a stable EL emission. The EL imaging allowed to identify the electroluminescent regions and the residual vacancy distribution associated with the fabrication technique. Measurements evidenced isolated electroluminescent spots where non-classical light emission in the 560–700 nm spectral range was observed. The spectral and auto-correlation features of the EL emission were investigated to qualify the non-classical properties of the color centers.

In the last decade diamond has gained increasing interest as a promising material for the development of efficient single-photon sources^1^,^2^, due to the discovery, the characterization and the integration in photonic structures of several luminescent centers associated with impurities and defects in its crystal matrix^3^,^4^,^5^,^6^,^7^. Their high quantum efficiency and stability at room temperature prefigure appealing applications in the emerging field of quantum communication^8^,^9^ as a competing candidate with respect to alternative platforms, such as quantum dots^10^ and silicon carbide^11^. In particular, the electrical stimulation of the lluminescence from a single-photon emitter by means of a controlled current injection would enable a straightforward development of solid-state opto-electronic devices, paving the way to integrated on-demand single-photon sources. The observation of electrically-stimulated photon emission in diamond was recently discussed in few works based on p-i-n junction devices, where emission from neutral nitrogen-vacancy (NV^0^) centers was reported, both in ensemble^12^ and as single-photon sources^13^,^14^, as well as from Xe-related^15^ and Si-V^16^ color centers ensembles. Particularly, the stimulation of non-classical electroluminescence (EL) required articulated device fabrication methods, relying either on the controlled homoepitaxial growth of suitably doped layers^13^ or on the co-implantation of P and B dopants^14^. The exploitation of scanning focused MeV ion micro-beams to directly define graphitic structures embedded in insulating diamond through the local introduction of radiation-induced structural damage^17^,^18^ offers an alternative strategy to simplify the fabrication process of charge-injecting electrodes in the bulk of the material. In particular, an ion fabrication technique relying on the strongly non-uniform damage profile of MeV ions to selectively graphitize buried layers in single-crystal diamond allowed the fabrication of particle detectors^19^, cellular biosensors^20^, surface acoustic waves generators^21^ and IR emitters^22^.

The above-mentioned works focused on the operation of the fabricated junctions at moderate voltage biases (i.e., ~30 V). On the other hand, the injection of charge carriers in an intrinsic diamond volume between two graphitic channels requires much higher electric fields than those applicable through standard surface electrodes. Such limitation is overcome by the fabrication of sub-superficial electrodes in the diamond bulk, where the extreme breakdown field of diamond can be fully exploited with this purpose.

In this work we report on a non-classical light emitting diamond-based device with sub-superficial graphitic electrodes for the electrical excitation of color centers. In particular, we show that such electrodes are suitable to provide a stable and non-destructive pump current for the stimulation of single-photon-emitting centers in the diamond bulk. Furthermore, the fabrication approach enabled for the first time the investigation of non-classical light electroluminescence from an electrical structure other than a p-i-n junction, providing an insight into the emission mechanism independently of the specific features of the device under investigation.

## Results

### The device

**Figure 1:**
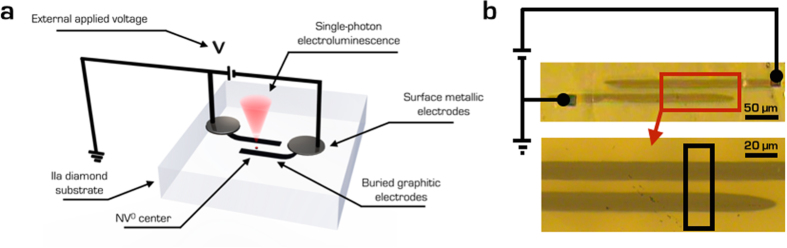
Overview of the single-photon electroluminescent device. (**a**) Two parallel buried electrodes are ion-microbeam-fabricated in single-crystal diamond and wire-bonded to an external voltage supply. The current flowing between the electrodes stimulates the electroluminescent emission from isolated color centers. (**b)** Optical micrographs of the device under investigation with a schematic representation of the electrical connections. The bottom image is a magnification of the area highlighted by the red rectangle.

The experiments were performed on a type-IIa single-crystal Element Six CVD diamond sample, denoted as “detector grade” due to its low nominal concentrations of substitutional nitrogen and boron (<5 ppb and <1 ppb, respectively). Two parallel sub-superficial graphitic microelectrodes were directly written in the diamond bulk by raster-scanning a ∅ ∼ 10 μm focused 6 MeV C^3+^ beam along linear paths. The ion fluence (~4 × 10^16^ cm^−2^) was high enough to overcome the graphitization threshold at the end of the ions range, thus ensuring the formation of amorphous microchannels at ~3 μm below the surface^17^. Subsequently, the sample was annealed in vacuum for 2 hours at 950 °C, with the purpose of both recovering the ion-induced residual structural damage in the regions surrounding the channels and converting the highly-damaged regions to a graphitic phase. In Fig. 1 a schematic of the fabricated microstructure and an optical micrograph of the sample are shown. The resulting device was structured with two independent ~10 μm wide and ~200 μm long parallel electrodes, with a spacing of ~10 μm.

**Figure 2:**
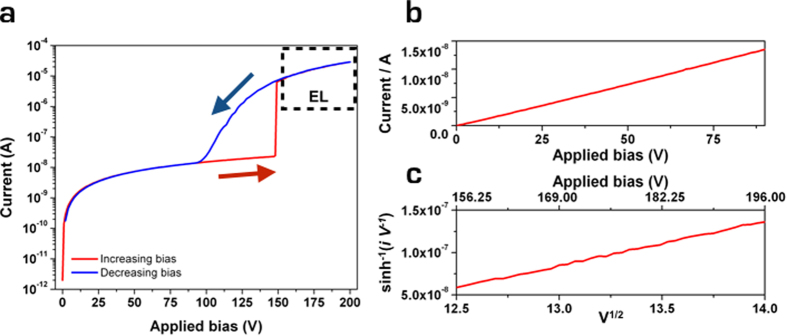
Charge-injection from sub-superficial electrodes. (**a)** *I*-*V* characteristic of the device. The red and blue lines indicate the curves acquired at increasing and decreasing voltage, respectively. (**b)** Plot in linear scale of the ohmic *I*-*V* behavior in the 0–95 V range. (**c)** Highlight of the PF behavior above the critical voltage *V*_*a*_. The quantities reported on the axes are chosen to linearize the PF expression *I* ∝ *V* sinh(*aV*^1/2^/*kT*).

A current-voltage (*I*-*V*) characteristic of the device is reported in Fig. 2. At increasing bias between 0 V and 150 V (Fig. 2a - red line plot, and Fig. 2b), a linear current increase (~15 nA at 100 V) is observed, indicating an ohmic conduction mechanism. As the voltage reaches the critical value *V*_*a*_ ~ 150 V, the system abruptly switches to a high-current regime, reaching ~30 μA at +200 V. When reverting the bias voltage back to zero (Fig. 2a - blue line plot), a broad hysteretic behavior is observed in the 100–150 V range, where higher currents are observed with respect to those measured at increasing bias. In the high-current regime (Fig. 2c), Poole-Frenkel (PF) conduction^23^ occurs, as described by the *I*-*V* trend: *I* ∝*V* sinh(*aV*^1/2^/*kT*).

**Figure 3:**
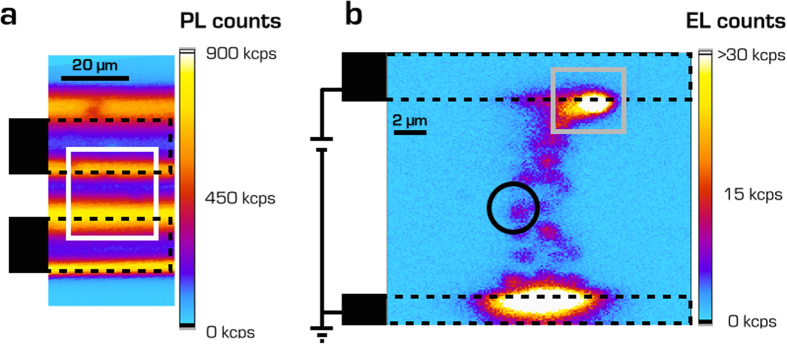
Mapping of luminescent emission. (**a**) PL map acquired with a λ = 532 nm laser excitation from the region highlighted by the black rectangle in Fig. 1b. (**b**) EL map (215 V bias) from the region highlighted by the white rectangle in Fig. 3a. The dashed black lines indicate the relative position of the electrodes.

EL occurred with the transition to the PF conduction regime, showing increasing intensity at increasing current, along a well-defined straight path (∅ < 5 μm) joining the graphitic electrodes (Fig. 3b), consistently with previous observations on field-emitting devices, reporting and modelling electron injection at localized sites at the interface between nanocrystalline diamond and graphite^24^,^25^. Such observations suggest that the carriers motion through trap states is thermally assisted by the impact of hot carriers injected in the diamond bulk, and are supported by the presence of a mixed phase at the interface between buried graphitic channels and single-crystal diamond^26^. The confinement of the current injection at a well localized position in the device is ascribed to a geometrical effect, e.g. the presence of a nanometric tip at the diamond/graphite interface, which is responsible for a local enhancement of the electric field.

At high bias voltages (150–215 V) the injected current was non-destructive over long operation times (>200 hours). The hysteretic behavior observed at decreasing bias was fully reproducible over multiple (>100) voltage cycles and was independent of the acquisition time (50–3000 ms range) of the electrometer, indicating the slow discharge of a space-charge field, associated with carriers detrapping in the inter-electrode areas, as pointed out in previous works^14^,^27^. Such interpretation is compatible with the presence of radiation-induced deep traps in the electrodes gap, due to the implantation of stray ions during the electrodes fabrication process.

### Non-classical light emission

The light emission properties were investigated by means of photoluminescence (PL) and EL mapping (Fig. 3), using a dedicated single-photon-sensitive confocal microscopy system^5^. In PL measurements, the device was unbiased and the color centers were excited with continuous laser light (*λ* = 532 nm, *P* = 0.4 mW). In EL measurements, the laser pump was replaced by the current injected in the inter-electrode gap. PL and EL maps were acquired at the same focal depth, i.e. ~3 μm below the diamond surface.

A typical PL map from a 20 × 60 μm^2^ region surrounding the buried graphitic electrodes is reported in Fig. 3a. The position of the electrodes is clearly visible as the map exhibits four bright horizontal bands, corresponding to their outer edges and indicating the presence of a large amount of radiation-induced color centers (B-band)^28^ formed by stray implanted ions during the fabrication process, as well as NV centers formed during the subsequent annealing. In Fig. 3b, an EL map acquired from the 17 × 17 μm^2^ area highlighted by the white rectangle in Fig. 3a is shown (bias voltage: 215 V). A short-pass filter (λ < 700 nm) removed the afore-mentioned B-band spectral component. The relative position of the buried electrodes is indicated by the dashed black lines. Two bright electroluminescent regions are clearly visible at the edges of the graphitic electrodes, which again can be attributed to higher concentrations of color centers due to the ion-beam microfabrication of the channels. In addition, several isolated spots in the inter-electrode gap region are aligned along a path joining the electrodes. The EL emission was stable over time, and maps acquired after operating the device for several tens of hours displayed the same results in both EL spatial distribution and intensity.

**Figure 4:**
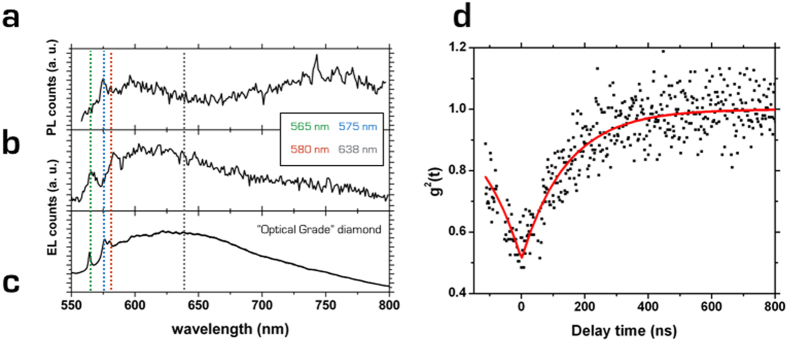
Non-classical EL emission. (**a**) PL (λ = 532 nm excitation) and (**b**) EL (240 V bias) spectra acquired from the bright spot in the gray square in Fig. 3b. (**c**) Reference EL spectrum acquired at 500 V applied bias from an “optical grade” device fabricated with a 6 MeV C^3+^ microbeam. (**d**) *C*_*N*_(*t*) curve acquired from the black circled spot in Fig. 3b. The curve is not corrected by background removal.

Both PL and EL (240 V bias voltage) spectra in the 550–800 nm range (Fig. 4a,b, respectively) were acquired from the bright spot at the edge of the top buried electrode (gray square in Fig. 3b) after removing all spectral filters. The PL spectrum highlighted the emission from NV^0^ centers, with a zero phonon line (ZPL) at λ = 575 nm and its phonon sidebands at higher wavelengths. Moreover, a broad emission in the 740–780 nm range, which can be attributed to the radiation damage-related B-band^14^,^28^ is clearly visible. The absence of significant NV^−^ emission could originate in principle from the low nitrogen concentration in the sample, determining a lack of donors that could modify the charge state of the defect^29^. Moreover, it has already been observed that the presence of a relevant concentration of acceptor-like radiation-induced defects^30^ or sp_2_ bonds^31^, as it is the case in the device under test, might promote the abundance of the NV^0^ charge state.

Also the EL spectrum is characterized by the absence of NV^−^ emission, more so because the center is not visible under electrical stimulation^13^,^14^,^28^. On the other hand, in the EL spectrum, a weaker but still noticeable emission in the 740–780 nm window is observed, confirming the attribution to the B-band, in agreement with previous EL measurements^14^. An additional peak is observed at λ = 565 nm, and it can ascribed to the ZPL of interstitial defects generated by MeV ion implantation and activated by the thermal annealing of the device^28^. Furthermore, the characteristic sidebands of the NV^0^ emission in the 590–640 nm spectral range are still observed. On the other hand, its ZPL emission at 575 nm is not visible, while an emission peak at λ ~ 580 nm is visible instead. It is worth noting that, although not discussed in details by the authors, a ~5 nm red-shift in the NV^0^ ZPL is visible also in Fig. 3c in ref. 14. However, additional ensemble measurements performed on an “optical grade” device (supplier: Element Six; substitutional N and B concentrations: <1 ppm and <0.05 ppm; equipped with graphitic electrodes fabricated by 6 MeV C^3+^ implantation; EL spectrum acquired at 500 V bias) indicate that this peak is associated with an additional interstitial-related ZPL, not active under 532 nm optical excitation and related to the 565 nm line (Fig. 4c)^28^,^32^.

Considering the spectral resolution of the monochromator, the ZPL of the NV^0^ center at 575 nm is thus arguably hidden by the intense emission at 580 nm. The acquisition of spectra from the isolated spots at the center of the EL map was not possible due to their low emission rates; however, the short-pass filter adopted for the EL map in Fig. 3b and the former spectral measurements allowed ruling out any potential contribution of color centers other than the ZPLs at 565 nm, 575 nm and 580 nm. It is worth noting that the integrated light emission associated with the 565 nm and 580 nm peaks is negligible with respect to the integrated emission of the NV^0^ center. However, while the non-classical emission discussed in the remainder of the text can be likely attributed to the NV^0^ center, an unambiguous attribution could not be determined under the given experimental conditions.

The isolated spot highlighted by the black circle at the center of the EL map in Fig. 3b was characterized in its non-classical emission properties. Second-order auto-correlation measurements were performed adopting a “Hanbury-Brown and Twiss” interferometry setup^6^. The normalized and background-uncorrected coincidence histogram *C*_*N*_(*t*) of the second-order auto-correlation function *g*^(2)^(*t*) (Fig. 4d) was evaluated. The curve displayed a minimum value at zero delay, highlighting non-classical photon emission. The *C*_*N*_(*t*) curve was qualified by means of a single-exponential function fit:


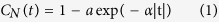


where the quantity 1 − *a* = *C*_*N*_(*t* = 0), representing the minimum value of the bunching dip at zero delay time, was evaluated as *C*_*N*_(0) = (0.51 ± 0.01). Considering that the detectors dark count rate is ~200 cps, this contribution alone results in a *g*^(2)^(*t*) noise correction to a value of 0.46, even without including EL background removal. The signal-to-noise ratio was evaluated as ρ = *S*/(*S* + *B*), where *S* is the actual EL signal emitted from the non-classical light source and *B* is the background ascribed to uncorrelated photons scattered from the surrounding luminescent regions. The value of ρ was estimated by imposing a single-photon emission at the zero delay time, i.e. ρ = (1−*C*_*N*_(0))^1/2^ = 0.7^14^,^33^,^34^. It is worth mentioning that, despite the low statistics, the value of ρ is in line with previous reports of ρ ~ 0.75^13^ (as estimated from the value of C_N_(0)) and ρ = 0.52^14^. Moreover, the absence of bunching components suggests that the emission photodynamics are substantially unaffected by the presence of a shelving state, as recently demonstrated for the NV^0^ center^34^.

The reciprocal of the fit parameter α in eq. (1), α^−1^ = (*R* + 1/τ) = (143 ± 5) ns describes the characteristic temporal width of the autocorrelation chronogram as the contribution of the current pump rate *R* and the center characteristic lifetime τ. Despite an extraction of the actual emission lifetime was prevented by the increase in the EL background at higher biases, the fit parameter indicates a lower efficiency of the electrical pumping with respect to the optical excitation of color centers, as found in previous works^13^,^14^,^15^.

An insight into the electrical stimulation mechanism occurring in a device ruled by a Poole-Frenkel conduction can be carried out assuming NV^0^ centers as responsible for the emission. In particular, while impact excitation is simply ruled out due to the lack of observed EL from the NV^−^ center, the electrical stimulation by exciton recombination^13^ at defect sites has also to be discarded, as this mechanism is likely quenched by trapping at the many states available in the band gap. Conversely, the separate capture of electrons and holes, as mentioned in ref. 13, still represents, on the basis of our results, a solid interpretational model. Particularly, the observed time scales of EL emission suggest that an explanation of the excitation process as the result of a charge state conversion of the center may not be implausible.

## Discussion

A single-crystal diamond device fabricated with sub-superficial graphitic microelectrodes was employed to provide an electrical stimulation of non-classical light emission from isolated diamond color centers. With respect to the previously reported fabrication processes, relying on the technologically challenging process of n-doping of diamond, the proposed technique presents the advantages of both fabrication simplicity and versatility. The technique could enable, in perspective, to define suitable electrodes geometries to electrically address deep color centers in diamond. In fact, the ion energy and species can be defined in order to finely align the electrodes within the diamond bulk with respect to the position of the single centers, purposely stimulating shallower or deeper defects with respect to the sample surface.

Furthermore, the exploitation of suitable implantation masks would enable the parallel fabrication of more elaborated electrodes arrays at specific positions of the device with sub-micrometric resolution^35^, leading to a significant reduction of the electrodes spacing and of the operating voltage. The employment of masks in the fabrication process could allow the exploration of novel three-dimensional geometries for the control of individual emitters in diamond.

In perspective, the technique would allow to define arrays and structures of electrically-stimulated single-photon sources in the diamond bulk at pre-selected positions, with a potential integration in optical circuits. Particularly, the device fabrication at few microns depth would enable the integration with surface photonic structure aiming at the increase of the light-emission efficiency, such as solid immersion lenses^36^ and nano-wires^37^.

## Methods

### Sample processing

after thermal annealing, the sample was oxidized in air for 30 min at a 400 °C and exposed to a 30 min oxygen plasma to remove surface conductivity caused by the sample graphitization and contamination occurred during the thermal treatment^6^. The electrical continuity of the sub-superficial channels with the sample surface was then ensured by exposing their endpoints to the sample surface by means of 30 keV Ga^+^ focused ion beam (FIB) milling. Care was taken to metal-mask the inter-electrode region during the FIB milling to avoid accidental Ga^+^ ion implantation. After a final cleaning step, 60 nm thick Ag contacts were deposited through a patterned contact mask at the surface-exposed endpoints of the channels to wire-bond the electrodes with the external circuitry.

### Confocal microscopy setup

In PL characterization the sample is kept at 0 V bias and the diamond color centers are excited with continuous light emitted by a solid-state laser source at λ = 532 nm (0.4 mW). The beam is focused on the sample through a 100× air objective (numerical aperture N.A. = 0.9). The sample is mounted on a remotely-controlled three-axis piezo-electric stage, with a scan area of 100 × 100 μm^2^ and 100 nm accuracy. The induced luminescence beam is collected together with the scattered excitation beam by the same focusing objective. A set of long-pass filters (λ > 650 nm) provides a suitable attenuation (>10^12^) of the laser excitation component of the beam. The filtered beam is then focused with an achromatic doublet and coupled into a graded-index multimode optical fiber, which both provides an optical connection to the detection system and acts as the pinhole aperture for the confocal system. The detection system consists of a photon-counter based on a Si-single-photon-avalanche photodiode (SPAD) operating in Geiger mode, with a dark count rate of ~200 cps. PL spectra were acquired removing the dichroic mirror and connecting the output of the multimode optical fiber to a computer-controlled single-grating monochromator (1200 grooves/mm blazed at 750 nm), with a spectral resolution of <5 nm FWHM. The monochromator output is then fibre-coupled and sent to another SPAD.

In EL measurements, the laser pump is replaced by the pump current between the buried electrodes. The long-pass filter is replaced by a short-pass filter attenuating light emission at λ > 700 nm, in order to discard the potential B-band spectral component. Spectral EL measurements are acquired removing all optical filters on the light path from the sample to the pinhole.

The “Hanbury Brown and Twiss” interferometry setup was based on a 50:50 beam-splitter integrated in the optical fiber, whose outputs are connected to two independent SPADs. A coincidence circuit, formed by a time-to-amplitude converter feeding a multi-channel analyzer, records a histogram of the photon coincidences as a function of the arrival time delay at the two SPADs.

## Additional Information

**How to cite this article**: Forneris, J. *et al.* Electrical stimulation of non-classical photon emission from diamond color centers by means of sub-superficial graphitic electrodes. *Sci. Rep.* **5**, 15901; doi: 10.1038/srep15901 (2015).
